# Dépistage sérologique de la maladie cœliaque chez des patients marocains atteints de diabète type 1

**DOI:** 10.11604/pamj.2016.24.103.8555

**Published:** 2016-05-31

**Authors:** Asmaa Drissi Bourhanbour, Sanae Ouadghiri, Nadia Benseffaj, Malika Essakalli

**Affiliations:** 1 Service de Transfusion Sanguine et d'Hémovigilance, Hôpital d'Enfants, CHU Rabat, UPR d'Immunologie, Faculté de Médecine et de Pharmacie de Rabat, Université Mohammed V Souissi, Maroc

**Keywords:** Maladie cœliaque, diabète type 1, dépistage sérologique, Celiac disease, type 1 diabetes, serological test

## Abstract

La maladie cœliaque (MC) est l'une des maladies auto-immunes les plus fréquemment associées au diabète de type 1 (DT1). La prévalence de MC dans DT1 varie de 3 à 6%. La présentation clinique de MC dans DT1 est classé comme asymptomatiques dans environ la moitié des cas. L'objectif de notre étude est de déterminer la fréquence des auto-anticorps anti-transglutaminase tissulaire (AtTG) et anti-gliadines (AAG) chez les patients diabétiques de type 1 dans le but de recommander une éventuelle biopsie jéjunale et d'instaurer un régime sans gluten précocement avant l'installation des signes cliniques et des complications de la maladie cœliaque. Les sujets inclus dans cette étude sont des patients atteints de DT1 non traités pour la MC et qui ne présentent pas de signes en faveur de cette pathologie. La détection des AtTG de classe IgG et IgA et AAG classe IgG et IgA a été réalisée par technologie Luminex. Nous avons inclus 31 patients. Il s'agit de 16 hommes et 15 femmes. Les AAG de classe IgA étaient positifs chez 4(13%) patients et chez 7(22,5%) patients pour les IgG. Les AtTG de classe IgA étaient positifs chez 3(10%) patients et chez une patiente (3%) pour les IgG. Dans notre étude l'association du diabète type 1 et des marqueurs biologiques de la MC n'est pas rare d'où l'intérêt de son dépistage systématique chez des diabétiques de type 1. Le diagnostic de cette forme atypique et silencieuse de la MC est important compte tenu du risque de complications sérieuses à type de malabsorption et de cancers digestifs.

## Abstract (en)

Celiac disease (CD) is an autoimmune disease frequently associated with type 1 diabetes (T1D). The prevalence of CD in patients with T1D varies from 3 to 6%. The clinical manifestation of CD in patients with T1D is classified as asymptomatic in about half of cases. Our study aims to determine the frequency of anti-tissue transglutaminase autoantibodies (IgA-tTG) and anti-gliadin antibodies (AGA) in patients with type 1 diabetes in order to early recommend jejunal biopsy and establish a gluten-free diet before the onset of clinical signs and complications of celiac disease. Subjects included in this study were patients with T1D and untreated CD who showed no signs of this disease. The detection of IgG tTG, IgG IgA and IgG AAG was performed using Luminex technology. We enrolled 31 patients. The study involved 16 men and 15 women. IgA AAG were positive in 4(13%) patients and IgG were positive in 7(22,5%) patients. IgA tTG were positive in 3(10%) patients and IgG was positive in one (3%) patient. In our study the association of diabetes type 1 with biomarkers of CD is not uncommon hence the importance of systematic screening for type 1 diabetes. The diagnosis of this atypical and silent CD form is important given the risk of serious complications such as malabsorption and gastrointestinal cancers.

## Introduction

La maladie cœliaque (MC) est une entéropathie auto-immune induite par l'exposition au gluten chez des sujets génétiquement prédisposés [1]. Elle représente l'une des maladies auto-immune les plus fréquemment associée au diabète de type 1 (DT1). En effet, elle survient chez les diabétiques de type 1 avec une prévalence variant de 3 à 6% [2] par rapport à une prévalence entre 0,7 et 2% dans la population générale [3]. Les auto-anticorps jouent un rôle important dans la pathogénie de la MC. Les peptides de la gliadine subissent une désamidation grâce à une enzyme intracellulaire ubiquitaire la transglutaminase tissulaire (tTG). La reconnaissance par le système immunitaire de la transglutaminase complexée à la gliadine entraîne la production d'auto-anticorps anti-transglutaminase (AtTG) et anti-gliadines (AAG). Les réactions immunitaire et inflammatoire ainsi induites provoquent la destruction de la muqueuse intestinale [4]. L'apparition de ces auto-anticorps précédent les signes cliniques et a donc un intérêt dans le dépistage de la MC. Les conséquences en termes de morbidité chez les patients cœliaques non traités, qui s'ajoutent à ceux du diabète rendent compte de la nécessité de poser un diagnostic précoce. En effet au cours du DT1, la MC est asymptomatique dans la moitié des cas [2]. L'objectif de notre étude est de déterminer la fréquence des auto-anticorps anti-transglutaminase tissulaires (AtTG) et anti-gliadines (AAG) chez les patients diabétiques de type 1 dans le but de recommander une éventuelle biopsie jéjunale et d'instaurer un régime sans gluten précocement avant l'installation des signes cliniques et des complications de la maladie cœliaque.

## Méthodes

Il s'agit d'une étude prospective réalisée auprès de patients suivis aux services d'endocrinologie du CHU Ibn Sina de Rabat au Maroc. Les sujets inclus dans cette étude sont des patients atteints de DT1 non traités pour la MC et qui ne présentent pas de signes en faveur de cette pathologie. La détection des anticorps AtTG et AAG a été réalisée par la technologie Luminex. C'est une technologie de cytométrie de flux avec deux lasers. Elle utilise comme support réactionnel des microsphères en polystyrène de 5,6 microns de diamètre. Ces microsphères sont marquées par deux molécules fluorescentes, et la combinaison de dix niveaux de concentration de chacun de ces deux colorants permet d'obtenir jusqu’à 100 types de microsphères différents. Chaque microbille, caractérisée par un code couleur, présente à sa surface un antigène différent. Ces microbilles sont mises en présence du sérum à tester et dans lequel la présence éventuelle d'auto-anticorps est recherchée. Après incubation, un conjugué fluorescent, permet de révéler et de quantifier la présence de ces auto-anticorps. Dans le cytomètre, chaque bille passe dans le faisceau de deux lasers. Un laser rouge qui identifie le code couleur de la bille par sa fluorescence intrinsèque et un laser vert qui mesure la quantité du conjugué fixée à la surface de la bille [5]. L'analyse des résultats a été faite par le logiciel ATHENA. Dans notre étude les sérums des patients sont testés sur des microbilles recouvertes de transglutaminases et de gliadines pour rechercher les auto-anticorps correspondants. Compte tenu de la fréquence non négligeable du déficit congénital en IgA dans la population générale, la recherche de ces même auto-anticorps mais d'isotype IgG a été faite systématiquement. La recherche des AtTG et AAG a été réalisé au niveau de l'unité d'auto-immunité du laboratoire d'immunologie du CHU Ibn Sina de Rabat.

## Résultats

Notre série comprend 31 patients dont 15 femmes et 16 hommes ce qui correspond à un sex ratio F/H de 0,94. Le dépistage des anticorps AtTG et AAG était positif chez 09 patients, soit 29%. Le Tableau 1 représente les principaux résultats de l’étude. Les AAG de type IgA étaient positifs chez quatre patients ce qui représente 13% des diabétiques. Ils étaient associés à une positivité des AAG de type IgG chez deux patients. Les patients qui avaient les AAG de type IgA négatifs ou douteux ont été dépisté pour les AAG de type IgG (déficit de l'IgA). Cinq patients étaient positifs pour les AAG de type IgG. Au total les AAG de type IgG étaient positifs chez sept patients, soit 22,5%. Les AtTG de classe IgG étaient positifs chez une patiente (3%) et chez trois (10%) patients pour les IgA.

**Tableau 1:** Nombre de patients diabétiques séropositifs pour la maladie cœliaque

Auto-anticorps (classe)	Patients séropositifs (N=31)
**AAG (IgA)**	**4**
**AAG (IgG)**	**7**
**AtTG (IgA)**	**3**
**AtTG (IgG)**	**1**

AAG: Anticorps anti-gliadine /AtTG : Anticorps anti-transglutaminasetissulaire

## Discussion

L'association de la MC et du DT1a été décrite pour la première fois en 1969 chez un enfant diabétique [6]. Depuis plusieurs études ont rapportés une incidence de plus en plus élevée et qui varie en fonction de la localisation géographique (Tableau 2). Dans notre étude, nous avons trouvé une séroprévalence des AAG et/ou AtTG de 29% chez les patients diabétiques de type 1. Ce chiffre est très élevée par rapport aux données de la littérature qui montrent que la prévalence de la MC dans le DT1 est 20 fois plus fréquente que dans la population générale et qu'elle varie en fonction des populations En Europe la prévalence sérologique varie entre 3.6% et 13.4% [7–10]. En Amérique elle est entre 8 et 11,8% [11, 12] et au Maghreb entre 4 et 16% [13–15]. La prévalence est significativement plus élevée dans les pays maghrébins liée à une fréquence élevée de la MC dans ces régions. Cependant de récentes études faites en Iran [16], Oman [17] et en Arabie saoudite [18] ont aussi mis en évidence des prévalences sérologique plus élevée de la MC chez les patients diabétiques (14.4%, 17% et 17.92%) (Tableau 2). Ces variations sont dues probablement à l'utilisation judicieuse de tests de dépistage sérologiques qui ont permis le diagnostic des formes silencieuses et pauci-symptomatiques. Au Maroc on ne dispose pas d’études de prévalence ni d’études multicentriques. La prévalence plus élevée dans notre échantillon nous pousse à étendre notre étude sur un nombre plus élevé de patients atteints de DT1 afin d'avoir une réelle estimation sur la prévalence de la MC dans le DT1 dans la population marocaine. Malgré, l'introduction de ces tests sérologiques de performance croissante (Tableau 3) et moins invasive, l'histologie duodénale typique (atrophie villositaire totale et sub-totale, lymphocytose intraépithéliale, hyperplasie des cryptes) demeure indispensable au diagnostic et à la qualification complète de la MC. Ce dernier examen fait défaut dans notre étude et sa réalisation pourrait réduire la prévalence de la maladie cœliaque dans notre échantillon.

**Tableau 2:** Prévalence de la maladie cœliaque chez les patients diabétiques dans la littérature

Origine géographique	Pays (N)	Prévalence Sérologique (n)	Références
**Europe**	**France (84)**	3,6%(53)	2004 [7]
	**Royaume-Uni (113)**	6.2% (7)	2007 [8]
	**Italie (8717)**	7,2%(629)	2011 [9]
	**Espagne (463)**	13.4% (62)	2010 [10]
**Amérique**	**Amérique du Nord (236)**	8% (19)	1998 [11]
	**Brésil (263)**	11,8% (31)	2006 [12]
**Maghreb**	**Tunisie (348)**	4% (14)	2005 [13]
	**Tunisie (205)**	8,3% (17)	2007 [14]
	**Algérie (116)**	16% (13)	1996 [15]
**Asie**	**Iran (83)**	14,4%(13)	2013 [16]
	**Oman (93)**	17% (16)	2013 [17]
	**Arabie Saoudite (106)**	17,92% (19)	2012 [18]

N: nombre d’échantillon; n: nombre de patients séropositifs

**Tableau 3:** Caractéristiques des tests sérologiques de diagnostic de la maladie cœliaque

	Sensibilité (%)	Spécificité (%)
**AGA IgA**	52-91	85-94
**AEM IgA**	97-100	98-99
**AtTG IgA**	95-99	75-94

AAG: Anticorps anti-gliadine /AtTG: Anticorps anti-transglutaminase tissulaire/AEM: anti-endomysium

## Conclusion

La MC et le DT1 sont deux affectations fréquentes associés à une morbidité et mortalité importante. Le risque accru de mortalité accompagnant la MC est attribué à des complications intestinales malignes tels le lymphome et l'adénocarcinome, dont le risque est respectivement de 6 et 1,3 fois plus fréquent que chez la population normale. De plus, près de 50% des malades sont exposés au risque d'ostéoporose. Ces complications peuvent être prévenues par le régime sans gluten, ce qui justifie le dépistage des formes peu symptomatiques, atypiques ou silencieuses. Au Maroc l'association MC et DT1 n’étant pas rare, le dépistage sérologique de la maladie cœliaque par la recherche des AAG et AtTG pourrait être instauré de manière systématique chez les patients DT1 dès le début de la maladie.

### Etat des connaissances actuelle sur le sujet

- Il est connu que la prévalence de la maladie cœliaque dans le diabète type 1 est 20 fois plus fréquente que dans la population générale et qu'elle varie en fonction des populations;
- Ces deux affections sont associées à une morbidité et mortalité importante;
- Cependant le dépistage de la maladie cœliaque chez les diabétiques n'est pas systématique.

### Contribution de notre étude à la connaissance

- A notre connaissance c'est la première étude qui s'intéresse à la prévalence de la maladie cœliaque chez les diabétiques type 1 marocains;
- Nous avons utilisé la cytométrie en flux pour la détection des anticorps.
